# Synthesis and Characterization of Silver Nanoparticles from *Rhizophora apiculata* and Studies on Their Wound Healing, Antioxidant, Anti-Inflammatory, and Cytotoxic Activity

**DOI:** 10.3390/molecules27196306

**Published:** 2022-09-24

**Authors:** Saeed Ali Alsareii, Abdulrahman Manaa Alamri, Mansour Yousef AlAsmari, Mohammed A. Bawahab, Mater H. Mahnashi, Ibrahim Ahmed Shaikh, Arun K. Shettar, Joy H. Hoskeri, Vijay Kumbar

**Affiliations:** 1 Department of Surgery, College of Medicine, Najran University, Najran 61441, Saudi Arabia; 2 Department of Surgery, Faculty of Medicine, King Khalid University, Abha 62529, Saudi Arabia; 3 Department of Pharmaceutical Chemistry, College of Pharmacy, Najran University, Najran 66462, Saudi Arabia; 4 Department of Pharmacology, College of Pharmacy, Najran University, Najran 66462, Saudi Arabia; 5 Division of Preclinical Research and Drug Development, Cytxon Biosolutions Pvt Ltd., Hubli-580031, Karnataka, India; 6 Department of Bioinformatics and Biotechnology, Karnataka State Akkamahadevi Women’s University, Vijayapura 586108, Karnataka, India; 7 Maratha Mandal’s Dental College and Central Research Laboratory, Belgaum 590019, Karnataka, India

**Keywords:** *Rhizophora apiculata* leaf, aqueous extract, silver nanoparticles, in vitro, wound healing, cytotoxicity, antioxidant, anti-inflammatory

## Abstract

Silver nanoparticles (AgNPs) have recently gained interest in the medical field because of their biological features. The present study aimed at screening *Rhizophora apiculata* secondary metabolites, quantifying their flavonoids and total phenolics content, green synthesis and characterization of *R. apiculata* silver nanoparticles. In addition, an assessment of in vitro cytotoxic, antioxidant, anti-inflammatory and wound healing activity of *R. apiculata* and its synthesized AgNPs was carried out. The powdered plant material (leaves) was subjected to Soxhlet extraction to obtain *R. apiculata* aqueous extract. The *R. apiculata* extract was used as a reducing agent in synthesizing AgNPs from silver nitrate. The synthesized AgNPs were characterized by UV-Vis, SEM-EDX, XRD, FTIR, particle size analyzer and zeta potential. Further aqueous leaf extract of *R. apiculata* and AgNPs was subjected for in vitro antioxidant, anti-inflammatory, wound healing and cytotoxic activity against A375 (Skin cancer), A549 (Lung cancer), and KB-3-1 (Oral cancer) cell lines. All experiments were repeated three times (*n* = 3), and the results were given as the mean ± SEM. The flavonoids and total phenolics content in *R. apiculata* extract were 44.18 ± 0.086 mg/g of quercetin and 53.24 ± 0.028 mg/g of gallic acid, respectively. SEM analysis revealed *R. apiculata* AgNPs with diameters ranging from 35 to 100 nm. XRD confirmed that the synthesized silver nanoparticles were crystalline in nature. The cytotoxicity cell viability assay revealed that the AgNPs were less toxic (IC_50_ 105.5 µg/mL) compared to the *R. apiculata* extract (IC_50_ 47.47 µg/mL) against the non-cancerous fibroblast L929 cell line. Antioxidant, anti-inflammatory, and cytotoxicity tests revealed that AgNPs had significantly more activity than the plant extract. The AgNPs inhibited protein denaturation by a mean percentage of 71.65%, which was equivalent to the standard anti-inflammatory medication diclofenac (94.24%). The AgNPs showed considerable cytotoxic effect, and the percentage of cell viability against skin cancer, lung cancer, and oral cancer cell lines was 31.84%, 56.09% and 22.59%, respectively. *R. apiculata* AgNPs demonstrated stronger cell migration and percentage of wound closure (82.79%) compared to the plant extract (75.23%). The overall results revealed that *R. apiculata* AgNPs exhibited potential antioxidant, anti-inflammatory, wound healing, and cytotoxic properties. In future, *R. apiculata* should be further explored to unmask its therapeutic potential and the mechanistic pathways of AgNPs should be studied in detail in in vivo animal models.

## 1. Introduction

The biosynthesis of AgNPs utilizing valuable medicinal plant extracts has expanded significantly due to the growing interest in studies of the nano-range of 1–100 nm for biomedical applications [1,2]. However, one of the essential components of modern nanotechnological techniques is the production of nanoparticles with an appropriate quality [1].

A vast array of the literature has described various strategies for metal nanoparticle synthesis involving chemical, electrochemical, photochemical, and other methods of reduction [3,4,5]. On the other hand, biological approaches have been claimed to be superior to chemical methods in terms of their economic feasibility and environmental safety. Micro-organisms’ biosynthesis of AgNPs is a well-established technique [6]. However, using plant-based materials rather than microbes for the green synthesis of metal nanoparticles has garnered much interest because of the lower toxicity, shorter processing time and the added benefit of natural capping agents. Furthermore, it lowers the cost of isolating microorganisms and improving culture conditions for microbe-assisted biosynthesis, and it employs a variety of reductant sources, including leaves, flowers and catkins [7].

AgNPs are primarily employed in antibacterial and anticancer treatments, but they are also used as biosensors, vaccine adjuvants, anti-diabetic agents, and in promoting bone and wound healing. High stability, high carrier capacity, the ability to incorporate both hydrophilic and hydrophobic molecules, and the ability to administer drugs via a variety of routes, including via oral application and inhalation, are key technological benefits of nanoparticles used as drug carriers. Excellent electrical conductivity, chemical stability, catalytic and antibacterial characteristics, as well as cytotoxic effects on cancer cells have all been demonstrated in silver nanoparticles.

At low concentrations, AgNPs have been shown to exhibit antibacterial, anti-biofilm and antiviral activities. Infection control is vital in medicine and various other industries, and a chemical-free option has become the need of the hour. Disinfection has become a vital parameter for pandemics such as COVID-19, and colloidal silver can be utilized as a novel standard for the preventative treatment of ventilator-acquired pneumonia in intensive care units of hospitals. Silver nanoparticles are effective antibacterial agents when used alone or in conjunction with existing medicines. After bioreduction by biosynthesized AgNPs, pharmaceutically important compounds can be preserved, which could have therapeutic implications. Because of their antibacterial properties, silver nanoparticles are commonly employed in the packaging industry to extend the shelf-life of food products [8,9,10].

Cancer is among the most significant causes of mortality around the globe, accounting for over 10 million deaths in 2020, or roughly one in every six. Cancers of the breast, lungs, gastrointestinal tract (including the cervix), and prostate are among the most prevalent. The five-year survival rate for oral, lung, and skin cancer (melanoma) is 55%, 26%, and 68%, respectively. The success rate of chemotherapy is not very encouraging, and many patients also experience severe side effects. Therefore, there is a pressing need to continue researching useful diagnostic and cutting-edge therapy targets of natural origin. Due to its lower toxicity, researchers have recently focused on creating/discovering novel therapeutic compounds using natural resources.

*Rhizophora apiculata* is one such mangrove plant, which belongs to the Plantae kingdom under the Rhizophoraceae family. It is well-known for its medicinal properties [11]. *R. apiculata* has medicinal properties in its roots, bark, and leaves. These trees are high in phytochemicals and have anticancer, antibacterial, antiemetic, antidiarrheal, and hemostatic activity [12,13]. Previously, *R. apiculata* has been assessed for its antibacterial activity [14], hepatoprotective activity [15], and cytotoxic activity [16,17]. Throughout the prehistoric era, plants were used as a source of treatment for various diseases worldwide. Numerous undiscovered natural products and nutrients have biologically beneficial properties [18]. As a result, there is an urgent need to discover and test new treatment alternatives that can effectively be used in disease management while causing minimal side effects. Hence, we thought it worthwhile to carry out this research with the following objectives: (1) phytochemical screening and quantification of secondary metabolites from *R. apiculata*, (2) green synthesis and characterization of silver nanoparticles from *R. apiculata*, (3) determination of in vitro antioxidant and anti-inflammatory activity of *R. apiculata* and its synthesized AgNPs, (4) in vitro anticancer activity of aqueous leaf extract of *R. apiculata* and its synthesized AgNPs against skin cancer, lung cancer, and oral cancer, and (5) in vitro wound-healing activity of aqueous leaf extract of *R. apiculata* and its synthesized AgNPs by scratch assay.

## 2. Results

### 2.1. Phytochemical Analysis

The findings of the phytochemical study showed that significant bioactive components such as glycosides, saponins, terpenoids, flavonoids, and phenols were present in the test extract (Table 1). The aqueous leaf extract of *R. apiculata* exhibited a total of 44.18 ± 0.08 mg/g of quercetin-equivalent flavonoid content and 53.24 ± 0.02 mg/g of gallic-acid-equivalent phenolic content.

### 2.2. Visual Observation and UV-VIS Characterization

**Figure 1:**
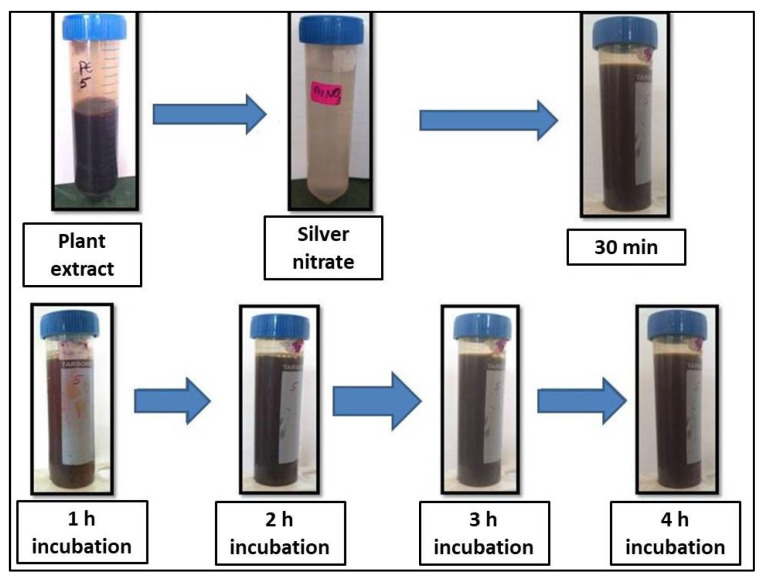
Formation of silver nanoparticles after adding aqueous leaf extract of *R. apiculata* with the change in time.

**Figure 2:**
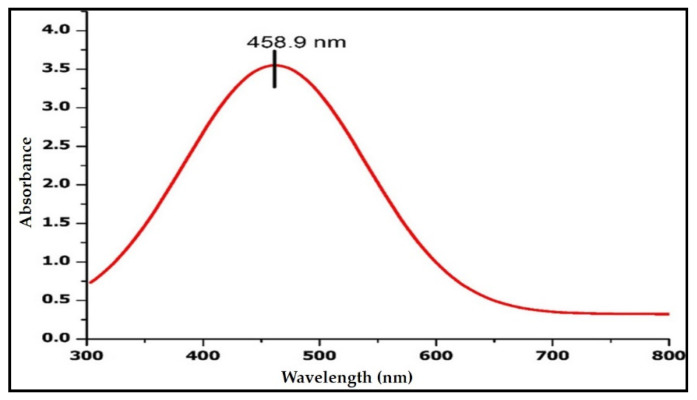
Silver nanoparticles were confirmed by UV-VIS spectral spectroscopy of colloidal solution.

The addition of *R. apiculata* leaf aqueous extract to 1 mM AgNO_3_ solution caused a color shift from yellowish to brown after 4 h, indicating the bioreduction of AgNO_3_ from silver metal ions (Ag+) to silver nanoparticles (Ag0) (Figure 1). After 1 h, the reaction was complete with a brown color. Silver nanoparticles were confirmed by UV-VIS spectral spectroscopy of the colloidal solution at 200–1100 nm. The maximal absorption peak (max) was at 459 nm (Figure 2).

### 2.3. SEM and EDX Studies

**Figure 3:**
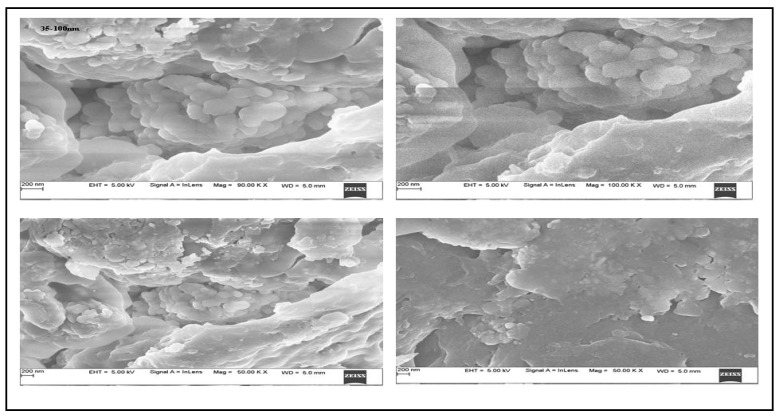
SEM images of *R. apiculata* AgNPs show an uneven shape and a diameter of 35–100 nm.

**Figure 4:**
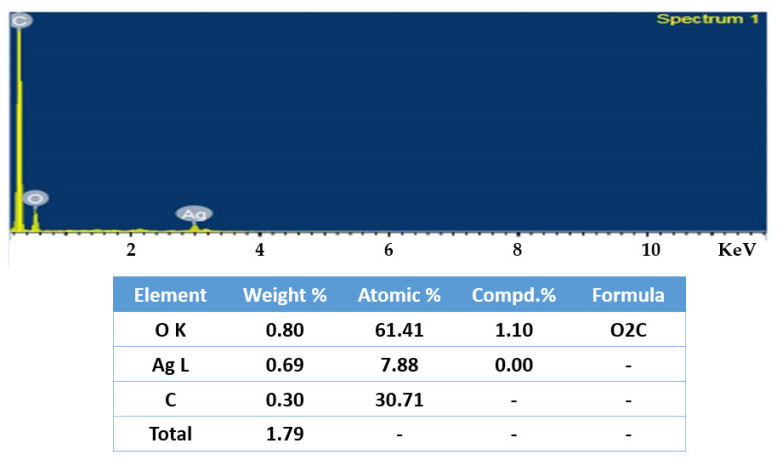
EDX analysis of *R. apiculata* AgNPs showing its elemental composition.

SEM investigations validated the morphology and surface of the AgNPs. The SEM images revealed silver nanoparticles with an irregular form and a diameter between 35 and 100 nanometers. EDX experiments helped to determine the purity and composition of the AgNPs. The formation of AgNPs was confirmed by the high signal in the 3 Kev metallic silver region. Besides silver, other elements including carbon and oxygen were found to make up 30.71% and 61.41%, respectively, of its elemental composition analyses (Figure 3 and Figure 4).

### 2.4. FTIR Analysis

**Figure 5:**
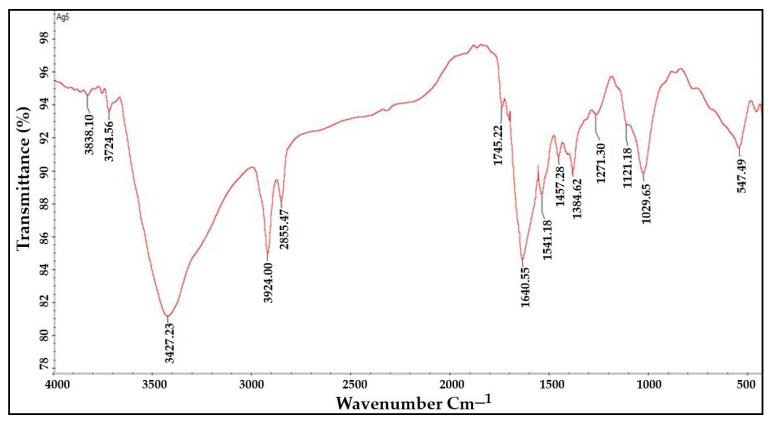
FTIR analysis of aqueous leaf extract of *R. apiculata* to determine its functional groups.

**Figure 6:**
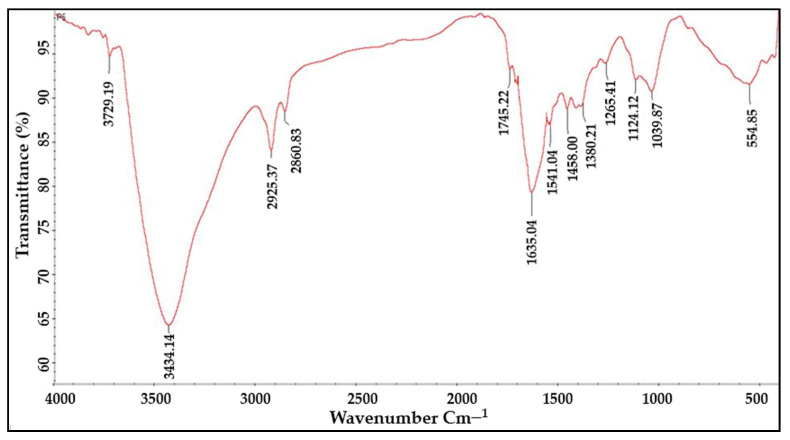
FTIR analyses of AgNPs synthesized from aqueous leaf extract of *R. apiculata* to determine functional groups responsible for the bioreduction.

The functional groups responsible for the bioreduction of Ag+ into Ag0 nanoparticles were identified using FTIR spectroscopy. It was found that functional groups such as aromatic and aliphatic amines, alkyl halides, alkynes, alcohols, esters, carboxylic acids, ethers, and nitrogen compounds were present in the infrared spectra of the plant extracts as well as the AgNPs (Figure 5 and Figure 6). The FTIR analysis of the AgNPs showed major peaks at 3729.19 cm^−1^, 3434.14 cm^−1^, 2957.37 cm^−1^, 2860.83 cm^−1^, 1745.22 cm^−1^, 1635.04 cm^−1^, 1541.04 cm^−1^, 1458.00 cm^−1^, 1380.21 cm^−1^, 1265.41 cm^−1^, 1124.12 cm^−1^, 1039.87 cm^−1^, and 554.85 cm^−1^ corresponding to the presence of alcohol, amines, imines, alkanes, nitro-compounds, phenols, and alkyl halides as a major functional groups. These functional groups might have been acting as capping, stabilizing, and reducing agents in the synthesis of nanoparticles from the plant extract.

### 2.5. Particle Size Analyzer and Zeta Potential

**Figure 7:**
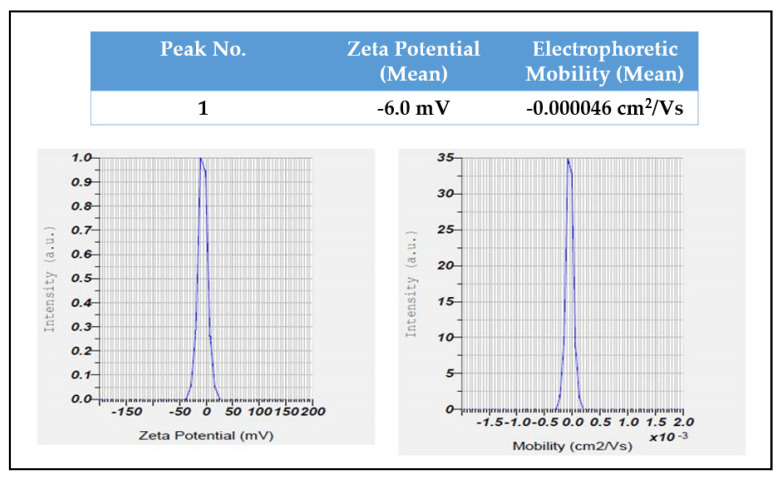
Zeta potential distribution of *R. apiculata* AgNPs.

**Figure 8:**
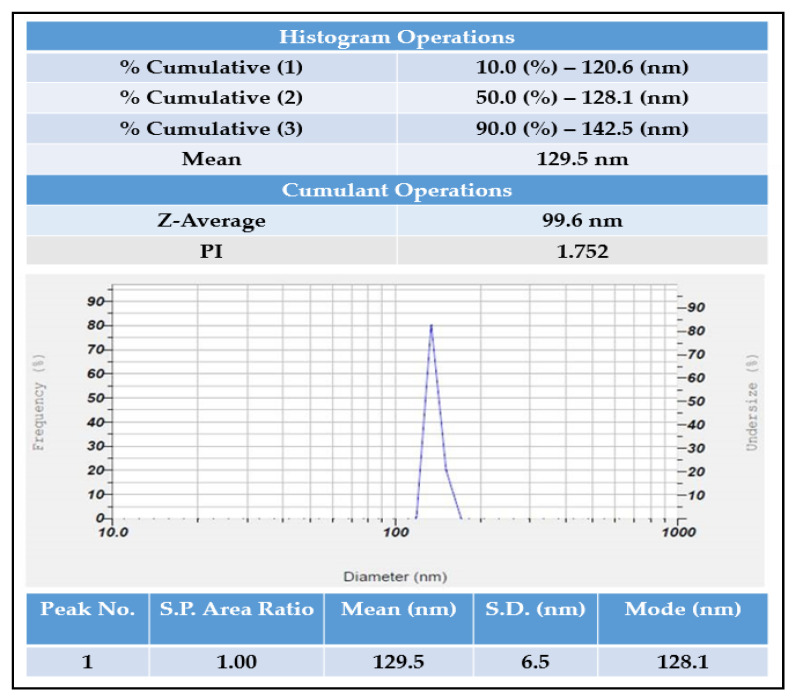
Particle size analysis of *R. apiculata* AgNPs.

Using a zeta potential analyzer with D.L.S., the average hydrodynamic size and dispersion of the AgNPs were analyzed. AgNPs with an average diameter of 99 nm, with a zeta potential of −6 mV, were found by the DLS analysis (Figure 7 and Figure 8).

#### XRD Analysis

**Figure 9:**
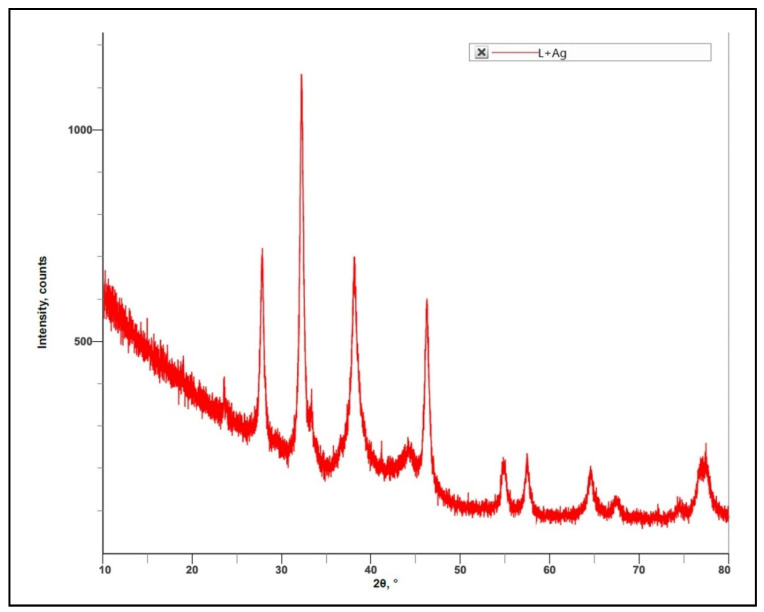
XRD analysis of *R. apiculata* AgNPs confirming its crystalline nature.

The XRD analysis showed sharp peaks at different angles i.e., 23.5210, 27.7920, 32.1954, 33.2915, 37.6234, 38.0849, 38.5761, 44.1340, and 46.1686. These degrees of angles corresponded to the crystalline nature of the material. Hence, it was confirmed that the synthesized silver nanoparticles were crystalline (Figure 9).

### 2.6. In Vitro Antioxidant Activity

The in vitro antioxidant activity of the synthesized AgNPs and plant extract was estimated using FRAP, H_2_O_2,_ DPPH, and PM assays.

#### 2.6.1. FRAP Assay

The FRAP assay was used to test different concentrations of the *R. apiculata* aqueous leaf extract and its AgNPs, with ascorbic acid as a standard. The antioxidant activity and absorbance increased when the concentration of both standard and AgNPs rose. The AgNPs had higher antioxidant activity than the standard, with an absorbance of 1.81 ± 0.02, while the standard showed an absorbance of 1.46 ± 003. The absorbance of the plant extract was 0.86 ± 0.02 (Table 2).

#### 2.6.2. H_2_O_2_ Assay

The hydrogen peroxide radical scavenging assay revealed that the AgNPs showed higher scavenging activity (74.98 ± 0.31% inhibition) than the standard (74.46 ± 0.13) and the plant extract showed a 63.58 ± 0.44% inhibition (Table 3).

#### 2.6.3. DPPH Assay

The *R. apiculata* leaf extract and its AgNPs were tested against ascorbic acid to determine their antioxidant potential. The aqueous leaf extract was shown to have equivalent antioxidant activity to the AgNPs, while the standard ascorbic acid exhibited the highest activity (Table 4).

#### 2.6.4. PM Assay

In this study, aqueous leaf extract of *R. apiculata* and its synthesized AgNPs were put through a PM assay along with the standard ascorbic acid. The AgNPs exhibited the highest antioxidant activity, followed by the aqueous plant extract and the standard ascorbic acid (Table 5).

#### 2.6.5. In Vitro Anti-Inflammatory Assay

A protein denaturation assay was used to test the anti-inflammatory activity of a known concentration of the AgNPs and aqueous leaf extract of *R. apiculata*. The anti-inflammatory effect of the extracts was comparable to that of the standard drug Diclofenac sodium. There were significant differences (*p* ≤ 0.001) in protein denaturation between the groups. The results showed that the standard drug had the highest anti-inflammatory activity (94.24 ± 1.90%), followed by AgNPs (71.65 ± 0.88%) and the plant extract (54.34 ± 3.26%) (Table 6).

### 2.7. Cytotoxicity Activity of Aqueous Leaf Extract of R. Apiculata and Its Synthesized AgNPs against Non-Cancerous Fibroblast L929 Cell Line

**Figure 10:**
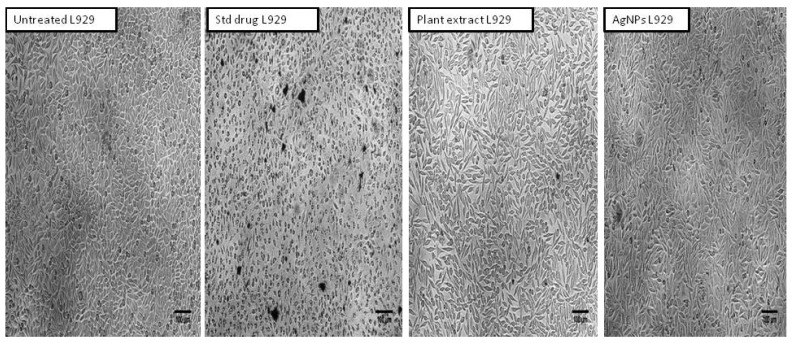
Morphological changes in L929 after treatment with aqueous leaf extract of *R. apiculata* and AgNPs.

The present cytotoxicity cell viability assay revealed that in both the tested samples, dose-dependent activity was observed. In comparison, the aqueous leaf extract of R. apiculata showed a larger decrease in the cell viability than the AgNP-treated cells. In the case of the plant extract, the percentage of cell viability was observed to be 46.81 ± 0.002%, whereas for the AgNPs, it was observed to be 77.50 ± 0.005 % (Table 7 and Figure 10).

### 2.8. Cytotoxic Activity of Aqueous Leaf Extract of R. apiculata and Its Synthesized AgNPs against A375 (Skin Cancer), A549 (Lung Cancer), and KB-3 (Oral Cancer)

**Figure 11:**
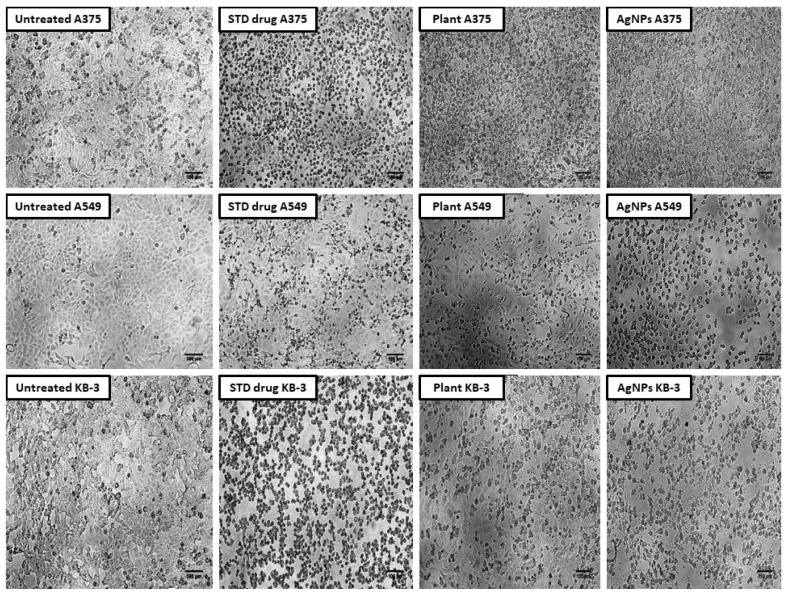
Morphological changes in cancer cell lines after treatment with aqueous leaf extract of *R. apiculata* and AgNPs.

The silver nanoparticles showed significant activity with a high percentage of cell death and a lower percentage of cell viability, i.e., for skin cancer (A375), the AgNPs showed 31.84 ± 0.004%, for lung cancer (A549) it was 56.09 ± 0.010%, and for oral cancer (KB-3) it was 22.59 ± 0.022%. On comparison within the tested group of cancer cell lines, the AgNPs showed a higher activity in oral cancer followed by skin cancer and lung cancer. In the case of the plant extract, the MTT cell viability results showed that, compared to the AgNPs, the extract showed moderate activity, i.e., for skin cancer (A375) 82.69 ± 0.002%, for lung cancer (A549) 73.73 ± 0.002%, and ovary cancer 67.17 ± 0.002% (Table 8 and Figure 11).

### 2.9. In Vitro Wound Healing Activity of Aqueous Leaf Extract of R. apiculata and Its Synthesized AgNPs

**Figure 12:**
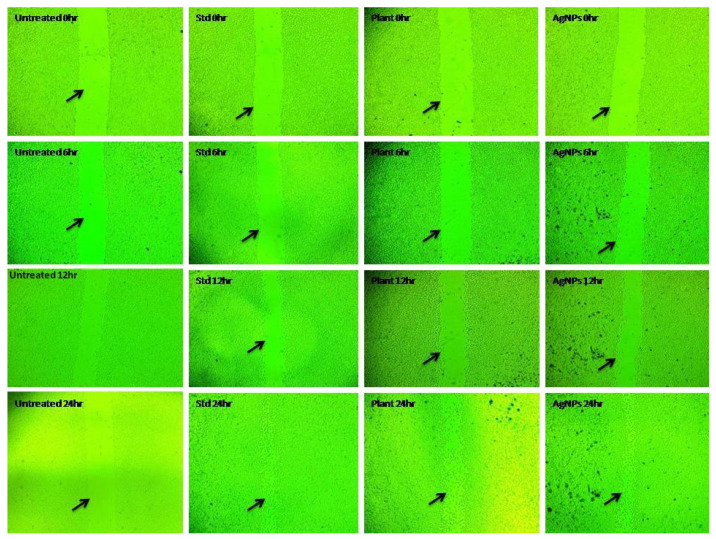
Microscopical images illustrating the ability of *R. apiculata* to heal wounds (in vitro). Images were taken at 0, 6, 12, and 24 h after mice fibroblast cells were cultured in the presence or absence of test and standard drugs.

The cell migration results showed an increase in the cell migration in the standard and AgNPs- treated cells than the aqueous leaf extract of *R. apiculata*. In comparison, the standard drug ascorbic acid showed higher cell migration activity followed by the AgNPs, plant extract, and the untreated group. For the standard at 6 h, 12 h, and 24 h, the cell migration was found to be 25.47 µm, 28.03 µm, and 21.74 µm, respectively. Similarly, the AgNPs showed 14.43 µm, 20.56 µm, and 18.23 µm of cell migration at 6 h, 12 h, and 24 h, respectively. The aqueous leaf extract of *R. apiculata* showed 11.63 µm, 14.58 µm, and 18.74 µm. In the case of the wound closure study, 24 hrs of incubation was considered for the analysis and calculation. In comparison, the standard drug ascorbic acid showed a value of 96.26% of wound closure. In contrast, the AgNPs and aqueous leaf extract of *R. apiculata* showed 82.79% and 75.23%, respectively, and for the untreated group, it was found to be 9.13% (Table 9 and Table 10 and Figure 12).

## 3. Discussion

The “green” method for making nanoparticles, which is faster than traditional chemical synthesis, is fascinating because it is good for the environment, saves money, is practical, and can be used in many different ways. Biosynthesized nanoparticles are a relatively new field, and they can be used in many different ways, such as drug delivery, cancer therapy, gene treatment and DNA analysis, antibacterial agents, biosensors, increasing response rates, and so on. Nanoparticles are used in electrical engineering, medicine, biology, textiles, and chemistry. The shape and size of colloidal metal particles are significant in many applications, such as making magnetic and electronic devices, healing wounds, expressing antimicrobial genes, and making bio composites. Based on the size and shape of the particles, the optical and electromagnetic properties of noble metal colloids vary [19].

Unlike physical and chemical methods, the green eco-friendly synthesis of nanoparticles is biocompatible, cost-effective, takes less time and energy, and is non-toxic. Biological sources such as plant extracts, bacteria, fungi, and algae are used to make silver nanoparticles [20,21]. This study used a method called “phytoreduction” to make silver nanoparticles from the *R. apiculata* plant. In the process of turning silver into silver nanoparticles, the leaf extract from *R. apiculata* was used as a reducing and capping agent. Screening for phytochemicals showed a wide range of secondary metabolites, most of which were saponins, glycosides, terpenoids, phenols, flavonoids, and tannins. The high levels of phenols and flavonoids in the aqueous leaf extract of *R. apiculata* were also quantified. For the creation of silver nanoparticles, a concentrated aqueous leaf extract was added to a solution of silver nitrate, and the change in the color confirmed the formation of AgNPs. After 4 h, the solution turned a dark brown color. The surface and shape of the AgNPs were confirmed by SEM studies. The SEM analysis revealed *R. apiculata* AgNPs with diameters ranging from 35 to 100 nm. The EDX analysis gives a qualitative and quantitative status of the elements that may contribute to the formation of N.P.s. EDX spectroscopy was used for elemental analysis. Based on the analysis, Ag seemed to be the key ingredient. Due to surface plasmon resonance (SPR) [22], metallic AgNPs show a typical optical absorption peak around 3.00 keV. A strong signal of metallic silver nanoparticles at 3 Kev confirmed the formation of silver nanoparticles. 

The FTIR spectra of the plant extract and AgNPs revealed the presence of functional groups such as alkyl halides, alkynes, aromatics and aliphatic amines, esters, ethers, alcohol, carboxylic acids, and nitro compounds, which may have acted as capping and reducing agents for the synthesis of the AgNPs. The FTIR findings were consistent with the findings of Rashid et al., 2021, who used FTIR analysis to determine the type of bonds and functional group in Au:ZnO (core:shell) nanoparticles via laser ablation nanoparticles prepared in deionized water [23].

The zeta potential method shows how the surface charge changes over time. This method is used to control the stability of metal nanoparticles in colloidal form. When the zeta potential of metal nanoparticles is either very positive or very negative, they tend to push away from each other and do not want to come together. However, when the absolute zeta potential is low, these particles stick together and clump together because no force keeps them from doing so. Using the dynamic light scattering measurement technique, the size of the silver nanoparticles was identified. Dynamic light scattering (DLS) is a way to measure the size of particles by pointing a laser beam on a suspension of particles or molecules moving in a Brownian motion. Size and zeta potential are important because they directly affect the particles stability, biodistribution, and uptake by cells [24,25]. In this study, a zeta potential analyzer with the DLS technique was used to figure out the average hydrodynamic size and size distribution of the green-made AgNPs. The DLS measurements showed that the AgNPs had an average diameter of 99 nm and a zeta potential of -6 mV. The amount of electrolyte and the pH of the dispersion have a big effect on the zeta potential of the particles. Due to negative repulsion, AgNPs have a good colloidal nature, stay stable over time, and spread out well when they have a high negative potential value [26].

Free radicals are chemical species with one or more electrons that are not paired with another electron. These are very unstable, and they take electrons from other molecules and damage them. These free radicals speed up the body’s abnormal, uncontrolled oxidation process. This causes the antioxidant defense system to fail and damages the cell structures, which increases the risk of Alzheimer’s, Parkinson’s, heart disease, liver disease, inflammation, and cancer, among other diseases. The risk of chronic disease and its progression can be reduced by boosting the body’s natural antioxidant defense or taking proven antioxidant supplements [27]. Antioxidant mechanisms in biological tissues are very complicated, and it is hard to judge the antioxidant power of crude extracts with just one method [28]. There are several in vitro testing methods for the antioxidant activity of pure compounds or extracts. So, in this study, four in vitro tests were used: the FRAP, PM, H_2_O_2_, and DPPH tests.

In the case of the FRAP assay, dose-dependent activity was observed. Among the tested groups, the silver nanoparticles showed the highest absorbance value, i.e., 1.81 ± 0.02 than standard ascorbic acid and aqueous leaf extract of *R. apiculata*. The standard drug ascorbic acid and the aqueous leaf extract showed 1.46 ± 0.003 and 0.86 ± 0.02, respectively.

The single concentration of silver nanoparticles, aqueous *R. apiculata* leaf extract, and standard ascorbic acid were tested for scavenging activity in a hydrogen peroxide assay. The AgNPs proved to be significant with the highest percentage of inhibition, i.e., 74.98 ± 0.31, whereas the standard ascorbic acid and aqueous leaf extract showed 74.46 ± 0.13 and 63.58 ± 0.44, respectively. 

In the DPPH assay, the results were expressed in terms of the percentage of inhibition. The AgNPs and aqueous leaf extract were compared with the standard drug ascorbic acid. The results proved that the aqueous leaf extract showed higher activity than the AgNPs and standard ascorbic acid with 83.91 ± 0.3 percentage of inhibition, whereas the AgNPs and standard ascorbic acid showed 76.74 ± 0.7 and 77.36 ± 0.3 respectively.

In the case of the PM assay, the antioxidant activity was measured and expressed in the form of absorbance. The AgNPs showed higher absorbance, i.e., 1.447 ± 0.004, whereas the standard ascorbic acid showed 1.131 ± 0.007 and aqueous leaf extract showed 1.27 ± 0.004.

Inflammation plays a big part in many diseases, such as atherosclerosis, arthritis, asthma, heart disease, cancer, and other diseases [29]. Steroids and non-steroidal anti-inflammatory drugs are the main types of medicines used to treat inflammation. Their associated side effects (GI problems and leukopenia, for example) have made it necessary to discover other anti-inflammatory drugs that work the same way but do not have any adverse effects [30,31]. A protein denaturation assay was used to test the anti-inflammatory activity of known concentrations (100 g) of synthesized AgNPs and aqueous leaf extract of *R. apiculata*. In vitro, the anti-inflammatory effect of the extracts was comparable to that of the standard drug, Diclofenac sodium.

The levels of cell viability and rates of cell growth are good ways to judge if a cell is healthy. Cell health and metabolism can be changed by physical and chemical things. These agents can be toxic to cells in different ways, such as by destroying cell membranes, stopping protein synthesis, binding irreversibly to receptors, stopping polydeoxynucleotide elongation, and stopping enzymes from carrying out their jobs [32]. In vitro cell viability and cytotoxicity assays are often conducted with cultured cells to test chemical safety and find new drugs. In recent years, there has been more interest in how these tests can be used. At the moment, these assays are also used in oncology research to test both the toxicity of a compound and its ability to stop tumor cells from growing. Because they are quick, cheap, and don’t need to use animals, their applications are growing in the research area.

In the present study, a toxicity assay was studied by using an in vitro MTT cell viability assay against a non-cancerous fibroblast L929 cell line. The cytotoxicity cell viability assay revealed that, in both tested samples, a dose-dependent activity was observed. On comparison, the aqueous leaf extract of *R. apiculata* showed a larger decrease in cell viability as compared to the AgNP-treated cells. In the case of the plant extract, the percentage of cell viability was observed to be 46.81 ± 0.002, whereas for the AgNPs it was observed to be 77.50 ± 0.005. The results from the present study corroborate with those of previously reported studies. For example, Wen et al., 2020, reported that *R. apiculata* silver nanoparticles had strong cytotoxic effects on human osteosarcoma MG-63 cells, which could be attributable to the silver nanoparticles’ antioxidant activity [16]. Similarly, Daphne et al., 2018, reported that AgNPs synthesized by potent yeast isolates demonstrated positive antioxidant and antibacterial action, with AgNPs being bacteriostatic at low concentrations (5 ug/mL) and bactericidal at high concentrations (100 ug/mL) [21].

AgNPs are superior to other bulk materials because they have a good structure, are stable, and have a larger surface area [33]. Physical and chemical routes of synthesis are hazardous compared to biological methods [34]. Recently, physical, chemical, radiation therapy, chemotherapy, and surgical treatments are not working as well as they used to for the management of cancer. A big reason why drugs do not work is that cancer cells become resistant to them [35]. The discovery and development of new drugs to stop the growth of cancer cells is challenging because of their side effects, toxicity, and high cost [36].

Most people with light skin develop skin cancer. In many developed countries, skin cancer cases have increased in the last few decades, which is a big health risk [37]. Therapeutic agents that are currently approved to treat malignant skin cancers have serious side effects, so it is important to come up with new therapeutic agents that work better and have fewer side effects [38,39]. Lung cancer is the most common type of cancer, and it kills and sickens many people all over the world. It is getting worse every day, and it is thought that about 10,000 new cases will happen every year [40]. In addition, it is the second most common non-communicable disease, with 50% of men and 30% of women having it [41,42].

In the present study, the cytotoxic activity was studied by taking different concentrations (10–50 µg) of aqueous leaf extract of *R. apiculata* and its synthesized AgNPs in A375 (Skin cancer), A549 (Lung cancer), and KB-3 (oral cancer) cell lines. Untreated and standard drug cisplatin-treated cells were taken as negative and positive groups. The silver nanoparticles showed significant activity with a high percentage of cell death and a lower percentage of cell viability in all the tested cell lines. On comparison within the tested group of cancer cell lines, the AgNPs showed a higher activity in oral cancer followed by skin cancer and then lung cancer. These results are consistent with previous studies, which reported a higher cytotoxic activity of the synthesized nanoparticles [16,24].

The skin is the body’s largest organ and is responsible for maintaining bodily homeostasis and protecting the body from pathogenic bacteria, UV radiation, and toxins, among other things [43]. Wounds form when the skin’s integrity is disturbed, and they usually recover in three months. Chronic wounds are defined as wounds that do not heal within three months for various reasons [44]. Chronic wounds, a silent killer, are one of the many risks that older people face. Non-healing chronic wounds affect not just elderly people, but also people with diabetes, nephropathies, cardiovascular diseases, and other lifestyle conditions [44]. A persistent wound that does not heal reduces an individual’s productivity and places a significant financial burden on the individual as well as the healthcare system [45]. The most commonly used medications to treat wounds in impoverished nations are cetrimide solution, sodium hypochloride, chlorhexidine, and others. These drugs have been shown to be ineffective and have negative effects when taken for long periods [46,47]. Burn patients are sensitive to infections because severe thermal injury damages the skin’s surface barrier and causes an immunological condition [48].

In the present study, the in vitro wound healing activity of aqueous leaf extract of *R. apiculata* and its synthesized AgNPs was studied by using scratch assay against a non-cancerous fibroblast cell line L929. The cell migration results showed an increase in the cell migration in the standard- and AgNP-treated cells than the aqueous leaf extract of *R. apiculata*. The standard drug ascorbic acid showed higher cell migration activity, followed by the AgNPs, the plant extract, and the untreated group. In the case of the wound closure study, the standard drug ascorbic acid showed 96.26% of wound closure, whereas the AgNPs and aqueous leaf extract of *R. apiculata* showed 82.79% and 75.23%, respectively, whereas for the untreated group it was found to be 9.13%. These results are consistent with the findings of Veeraraghavan et al., 2021, who reported that silver nanoparticles from aqueous extract of *Scutellaria barbata* had potent wound healing activity confirmed with a wound scratch assay on fibroblast cells (L929) [49]

The emerging biochemical applications of nanotechnology are focused on the development of nanoparticles with enhanced antioxidant and antibacterial properties to combat degenerative diseases such as Alzheimer’s and cancer, among others [50,51]. Due to their unique physiochemical properties, metal nanoparticles have received a lot of attention and are now a hot topic for research in fields such as sensors, imaging, cosmetics, cancer therapy, and drug delivery [52]. When silver nanoparticles were invented, they altered the way silver was used in the medical industry [44]. Increasingly, silver nanoparticles are being used in wound dressings, medicine transporters, and even in artificial implantations because of their antibacterial properties [53,54].

AgNPs toxicity to human tissues has been studied extensively in recent years, and it has been found safe for medicinal formulations [55]. A recent review has highlighted the applications of AgNPs in the pharmaceutical and cosmetic industry, tissue engineering, anti-infective therapy and wound care, food, and the textile industry [56]. The present study’s findings are consistent with those of previous studies that have reported the various biomedical applications of AgNPs [57,58,59,60]. As a result, nanotechnology is significantly advancing healthcare field. Researchers use nanoparticles in drug delivery systems, medical imaging, and tumor targeting. Nanoparticle-based medicines have several uses; they can both detect cancers and transport drugs to treat them [58,59].

## 4. Materials and Methods

### 4.1. Collection of Plant Material

The plant material (leaves) was collected from the Anshi jungle region of the Western Ghats, in Uttar Kannada District, Karnataka State, India, in March 2022, and was identified by Dr. Kotresha K, of Karnataka Science College’s Department of Botany, Dharwad, Karnataka, by consulting the voucher specimen that is deposited in Karnataka Science College, Dharwad, Karnataka. The leaves were shade-dried, and a motorized grinder was used to reduce the dried leaves into a coarse powder. The powder was maintained in sealed containers at room temperature for further use. All the chemicals and solvents used in this research were purchased from Hi-media in India and were of analytical quality.

### 4.2. Preparation of Plant Extract

For 48 h, a Soxhlet device was used to extract 25 gm of powdered leaves in 1000 mL of distilled water. The above procedure was repeated for four batches, i.e., 4000 mL of distilled water and 100 gm of powdered leaves were used for the extraction process. The resulting aqueous extract was concentrated using a rotary evaporator. The subsequently concentrated extract was then dried in desiccators to give a final yield of 3.8 g of dried extract and kept in an airtight bottle at 4 °C for further use. The percentage yield was calculated using the following formula: % Yield = R/S 100, where R = extracted weight by leaf residue and S = initial sample weight. AgNPs were synthesized using the *R. apiculata* aqueous extract as a reductant and stabilizer [61].

### 4.3. Qualitative Analysis of Metabolites

Following the procedure described by Deepti et al. (2012): the crude aqueous leaf extract of *R. apiculata* was qualitatively tested for the presence of various groups of metabolites, including flavonoids, alkaloids, phenols, glycosides, lignins, sterols, saponins, anthraquinones, tannins, and reducing sugars [62].

### 4.4. Estimation of Total Phenolic Content

Spectrophotometry was used to determine the total phenolics in the plant extract (Singleton et al., 1999) [63]. Calibrating curves for gallic acid (G.A.) were made in the range of 20–100 µg/mL. Lastly, the phenolic concentrations were changed into gallic acid equivalents, i.e., µg GA/g of dry weight (dw) of the extract. The standard calibration curve (Y = 0.001X + 0.113) was used to determine the total amount of phenolics.

### 4.5. Estimation of Flavonoids Content

The amount of flavonoids present in the plant extract was determined using the method described by Chang et al., 2002 [64] using quercetin as a reference standard, and the absorbance of the sample was quantified at 415 nm using a UV-VIS spectrophotometer.

### 4.6. Synthesis of Silver Nanoparticles

A total of 1 mL of *R. apiculata* leaf extract (concentration 50 mg/mL) was added to 10 mL of 1 mM AgNO_3_ aqueous solution (ratio 1:10) and shaken vigorously for 30 s. The complete reaction mixing procedure was conducted in a dark environment at room temperature (26–27 °C) to minimize unwanted photochemical reactions. The oxidation/reduction process was visible after the colorless reaction mixture had been incubated and reacted for the required time [65]. An aqueous mixture containing AgNPs was centrifuged at 10,000 rpm for 10 min and then re-dispersed in double-distilled water to remove the remaining aqueous extract from the freshly synthesized AgNPs, which were allowed to dry in powder [66]. The AgNP synthesis was performed on a pilot scale to optimize the conditions. Later on, the process was scaled-up for the synthesis of *R. apiculata* AgNPs in a single batch.

### 4.7. Characterization of Newly Synthesized AgNPs

All of the characterizations and bioassays performed in this investigation used a single batch of the synthesized *R. apiculata* AgNPs. An array of analytical methods were employed to characterize the produced AgNPs. These included FTIR and U.V.–visible analysis; energy dispersive X-ray analysis; scanning electron microscopy; zeta potential, and particle size analyzer.

#### 4.7.1. U.V.–Visible Spectroscopy-Based Analysis

To confirm the colloidal solution’s reduction in silver ions, a U.V.–visible spectroscopy (U-3310, Hitachi, Tokyo, Japan), was used to examine the U.V. spectrum of 1 mL of an aliquot sample in a quartz cuvette, scanning for wavelengths between 200 and 700 nanometers, in comparison to distilled water as a control and 1 mM AgNO_3_ as a blank [67].

#### 4.7.2. FTIR-Based Analysis

To identify the functional groups (bio-groups) that bind to the silver surface and play a role in the creation of AgNPs, FTIR spectroscopy was utilized (S700, Nicolet, MA, USA). After 72 h of incubation, the reaction mixtures were centrifuged three to four times at 10,000 rpm for 15 min to separate the AgNPs. Deionized water was used to replace the supernatant, and the particles were powdered and stored. The dried AgNPs were pelleted with potassium bromide in 1:100 ratios and submitted to FTIR analysis [68,69].

#### 4.7.3. Scanning-Electron-Microscopy-Based Analysis

Scanning electron microscopy (JSM-IT 500, Jeol, Boston, MA, USA), was used to determine the surface morphology of nanoparticles. After a 4- to 6-h reaction, the sample was prepared by centrifuging a colloidal solution at 10,000 rpm for five minutes. The pellet was re-centrifuged and re-dispersed in deionized water, a procedure carried out numerous times before the dry pellet was obtained. Once the AgNPs had been cleaned, they were suspended using sonication for 5–10 min, one cycle at a time. Carbon-coated grids were then used to set the suspension’s drop on. The sample was entirely dried under light. SEM analysis was performed on the prepared sample [69].

#### 4.7.4. Energy Dispersive X-ray

In this study, reduced AgNPs were dried on a carbon-coated copper grid, then exposed to EDX (JSM-IT 500, Jeol, Boston, MA, USA), and the elemental composition of the reduced AgNPs was determined using this approach [68].

#### 4.7.5. Zeta Potential Observations of N.P.s

The zeta potential of the colloidal N.P.s may be employed to gather further information on their stability. The zeta potential amplitude suggests the possible colloid stability based on its measurement. According to Meléndrez and colleagues (2010) [70], particles with zeta potentials more positive than +30 mV or more negative than −30 mV are normally considered stable. With the laser zeta meter, we examined potentials on the surface (Malvern zeta seizer 2000, Malvern, Worcestershire, UK). NaCl was used as a suspending electrolyte solution (2 10–2 M NaCl) to dilute the liquid nanoparticle samples (5 mL). After that, the pH was set to the desired level. For 30 min, the samples were shaken vigorously. Metal particles’ zeta potential and equilibrium pH were obtained after shaking. The silver nanoparticles’ surface potential was measured using a zeta potential analyzer. Each measurement was based on an average of three measurements.

#### 4.7.6. Particle Size Analyzer

The PSA analysis was performed on a lyophilized and ultrasonicated sample (SZ-100, Horiba, Kyoto, Japan) to determine the samples’ particle sizes [68].

#### 4.7.7. X-ray Diffraction (XRD) Analysis

In the present study, the synthesized silver nanoparticles from aqueous leaf extract of *R. apiculata* were subjected to XRD analysis (Smart Lab SE, Rigaku, Tokyo, Japan) to determine the nature and average size of the nanoparticles [23].

### 4.8. In Vitro Measurement of Antioxidant Activity

#### 4.8.1. Ferric Ion-Reducing Antioxidant Power Assay (FRAP)

According to Oyaizu. (1986) [71] with a minor adjustment, the ferric ions’ reducing power was assessed. Concentrations of aqueous extract of *R. apiculata* leaf and the synthesized AgNPs ranged from 100, 200, 300, 400, and 500 µg/mL. The mixture was then incubated for 30 min at 50 degrees Celsius with 2.5 milliliters of 20 mM phosphate buffer and 2.5 milliliters of 1 percent *w*/*v* potassium ferricyanide. It was then cooled and incubated for another 10 min before adding 2.5 mL trichloroacetic acid and 0.5% ferric chloride to the mixture. A UV-VIS spectrophotometer was used to detect the absorbance at 700 nm. Ascorbic acid was utilized as a benchmark. Each sample was tested three times in triplicates.

#### 4.8.2. Hydrogen Peroxide Scavenging Assay

*R. apiculata* leaf extracts and their generated AgNPs were tested for their capacity to scavenge hydrogen peroxide using ascorbic acid as a reference to evaluate their antioxidant activity. A total of 0.6 mL of phosphate buffer (pH-7.4) containing 4 mM H_2_O_2_ was added to 0.5 mL of standard ascorbic acid at a known concentration, as well as to tubes containing plant extracts at various concentrations ranging from 100 to 500 µg/mL in phosphate buffer (pH-7.4). We assessed the solution’s absorbance at 230 nm after ten minutes using a phosphate buffer and a hydrogen-peroxide-free blank solution. Phosphate buffer was substituted for the sample or standard in the control preparation. Each sample was tested three times. The formula approach was used to compute the percentage of inhibition.

Percentage of inhibition % = A_c_ − A_t_/A_c_ × 100

#### 4.8.3. DPPH Free-Radical-Scavenging Ability Assay

The free-radical-scavenging activities of the *R. apiculata* aqueous leaf extract and its produced AgNPs were evaluated using the 1,1-diphenyl-2-picrylhydrazyl (DPPH) free radical test [72]. A total of 3 mL of 0.1 mM DPPH in methanol solution was added to each aqueous extract and its AgNPs. Tubes were vigorously shaken and left at room temperature for 30 min in the dark. At 517 nm, absorbance was measured using a UV-VIS spectrophotometer. The control was distilled water, and the standard was ascorbic acid. Following the below formula, each sample’s ability to scavenge DPPH was determined.

% inhibition = A_c_ − A_t_/A_c_ × 100

where in A_c_ is the absorbance of the control and A_t_ is the absorbance of the test sample. Experiments were carried out in triplets. All of the substances that were tested had their IC_50_ values determined. A higher level of free radical activity was indicated by lower absorbance of the reaction mixture.

#### 4.8.4. Phosphomolybdenum (PM) Assay

According to Prieto et al., 1999 [73], the standard PM test was used to determine the total antioxidant activity. Leaf extracts and the AgNPs were added to each test tube at a concentration of 100, 200, 300, 400, and 500 µg/mL, and each tube included 3 mL of distilled water and 1 mL of molybdate reagent. For 90 min, these tubes were held at 95 °C. A 695 nm absorbance reading was taken after the reaction mixture was adjusted to room temperature for 20–30 min. Ascorbic acid was utilized as a standard.

### 4.9. Evaluation of In Vitro Anti-Inflammatory Activity

Using the protein denaturation technique published by Padmanabhan et al., 2012 [74], the anti-inflammatory efficacy of *R. apiculata* aqueous leaf extract and its AgNPs was investigated. The potent non-steroidal anti-inflammatory drug diclofenac sodium was used as a standard drug. A total of 2 mL of synthesized AgNP (100 μg/mL) was added to 2.8 mL of phosphate-buffered saline (pH 6.4) and 2 mL of egg albumin (from fresh hen’s egg (1 mM)) in the reaction mixture, which was incubated for 15 min at 27 ± 1 °C. The mixture was heated in a water bath at 70 °C for ten minutes to produce denaturation. After cooling to room temperature, the absorbance was measured at 660 nm. Each experiment was carried out three times over. To calculate the percentage of protein denaturation inhibition, the following formula was used:

% inhibition= A_c_−A_t_/A_c_ × 100

where, A_t_ = absorbance of test sample; A_c_ = absorbance of control.

### 4.10. Determination of the Cytotoxic Activity of Aqueous Leaf Extract of R. apiculata and Its Synthesized Silver Nanoparticles Using MTT Assay

A standard colorimetric MTT assay using 3-(4,5-dimethylthiazol- 2-yl)-2,5-dimethyl tetrazolium bromide dye (Sigma, St. Louis, MO, U.S.A.) was used by Carmichael et al. [75] to test the effect of *R. apiculata* aqueous leaf extract and synthesized silver nanoparticles on the viability of non-cancerous fibroblast cells L929 (1987). Based on dose–response curves created for each cell line, an IC_50_ value for the concentration of the test medication needed to suppress cell growth by 50% (IC_50_) was computed. Mitochondrial dehydrogenase of undamaged cells reduces MTT to a purple formazan residue [76].

Inhibition Percentage = OD of Test sample ÷ OD of control × 100

### 4.11. In Vitro Wound Healing Study by Using Scratch Assay Test

Plant extract and silver nanoparticles were examined for the migration capabilities of the L929 cell line [77]. Animal cell culture plates with DMEM medium supplemented with 10% FBS and 2 percent penstrip antibiotic were used to start the cell culture process. After achieving a cell density of roughly 50,000, a sterile 100 μL plastic pipette tip scratch was used to generate a monolayer confluent of cells. PBS solution was used to wash away cell debris. Untreated cells were used as a negative control, whereas standard ascorbic acid was used as a positive control. A 24-h incubation period at 37 °C with 5% CO_2_ was then carried out on the cells. To evaluate relative cell migration and wound closure, the scratched cell layers were incubated for various lengths of time: 0 h, 6 h, 12 h, and 24 h. MagVision Software was used to calibrate 4× magnification to assess the gap distance. Applying the following formula, wound closure and migration rates were calculated:

Wound closure (%) = A_0h_ − A_Th_/A_Th_ × 100

Rm (Rate of migration) = W_i—_W_f_/*T*

where A_0h_ represents the wound’s initial size, A_Th_ represents the wound’s size after h hours, Rm represents the rate of migration (m/h), Wi represents the initial wound width (μm), W_f_ represents the final wound width (μm), and *T* represents the duration of migration (hour).

### 4.12. Statistical Analysis

All experiments were conducted in triplicate (*n* = 3), and the results were reported as the mean standard deviation and the standard error of the mean. Using a one-way ANOVA analysis in the IBM SPSS Version-20, we determined the differences in means between the various groups.

## 5. Conclusions

The present study is one of the first to report the *R. apiculata* AgNPs wound healing potential and cytotoxic activity against the A375 (Skin cancer), A549 (Lung cancer), and KB-3-1 (Oral cancer) cell lines. In the present study, aqueous leaf extract of *R. apiculata* was used as a reducing agent for the synthesis of silver nanoparticles from silver nitrate. The synthesized silver nanoparticles were characterized by several techniques such as SEM-EDX, FTIR, a particle size analyzer, XRD, and zeta potential. All the analytical techniques confirmed the synthesis of silver nanoparticles. Antioxidant, anti-inflammatory, and cytotoxicity investigations against cancer cell-lines revealed that the AgNPs had significantly higher activity than the plant extract. In the case of the in vitro wound healing activity, the AgNPs showed more potent cell migration and wound closure than the plant extract. In future, *R. apiculata* should be further explored to unmask its therapeutic potential and the mechanistic pathways of AgNPs should be studied in detail in in vivo animal models. In light of these encouraging findings, we recommend further research into the following areas: the effect of harvesting location, season, and quantity on metabolite, phenolic, and flavonoid presence; the feasibility of performing at least one additional scale-up synthesis of an additional extract to demonstrate effects similar to those reported in the manuscript; and the stability of extracts and nanoparticles during storage to ensure its safety.

## Figures and Tables

**Table 1:** Phytochemical Analysis of aqueous leaf extract of *R. apiculata*.

Tests	Water Extract
Alkaloids	−
Flavonoids	+
Glycosides	+
Phenols	+
Saponins	+
Tannins	−
Terpenoids	+
Steroids	−

+: present; −: absent.

**Table 2:** FRAP assay of aqueous leaf extract of *R. apiculata* and its synthesized AgNPs.

Sl.No	Concentration (µL/mL)	Optical Density at 700 nm		
		Std Ascorbic Acid	*R. apiculata* Extract	AgNPs
1	100	0.46 ± 0.003	0.44 ± 0.003	0.60 ± 0.036
2	200	0.75 ± 0.004	0.48 ± 0.003	0.80 ± 0.036
3	300	0.99 ± 0.004	0.53 ± 0.005	1.03 ± 0.054
4	400	1.13 ± 0.004	0.63 ± 0.003	1.51 ± 0.017
5	500	1.46 ± 0.003	0.86 ± 0.025	1.81 ± 0.025 *

Results were performed in triplicates and expressed as mean ± standard error. * One-way ANOVA followed by post Tukey’s test revealed statistically significant differences (*p* ≤ 0.001) between the groups.

**Table 3:** H_2_O_2_ assay of aqueous leaf extract of *R. apiculata* and its synthesized AgNPs.

Sl. No	Concentration (µg/mL)	Samples	Percentage (%) Inhibition
1	100	Standard	74.46 ± 0.13 *
2	100	*R. apiculata* extract	63.58 ± 0.44 *
3	100	AgNPs	74.98 ± 0.31 *

Results were performed in triplicates and expressed as mean ± standard error. * One-way ANOVA followed by post Tukey’s test revealed statistically significant differences (*p* ≤ 0.001) between the groups.

**Table 4:** DPPH assay of aqueous leaf extract of *R. apiculata* and its synthesized AgNPs.

Sl.no	Concentration (µg/mL)	Percentage Inhibition		
		Std Ascorbic Acid	*R. apiculata Extract*	AgNPs
1	10	67.78 ± 0.17	43.56 ± 0.70	51.04 ± 1.42
2	20	73.65 ± 0.23	46.27 ± 0.23	55.34 ± 0.98
3	30	78.20 ± 0.30	63.64 ± 0.35	63.48 ± 1.33
4	40	80.80 ± 0.35	72.9833 ± 0.66	66.39 ± 0.70
5	50	83.91 ± 0.35	77.36 ± 0.35	76.74 ± 0.76 *

Results were performed in triplicates and expressed as mean ± standard error. * One-way ANOVA followed by post Tukey’s test revealed statistically significant differences (*p* ≤ 0.001) between the groups.

**Table 5:** PM assay of aqueous leaf extract of *R. apiculata* and its synthesized AgNPs.

Sl.no	Concentration (µg/mL)	Optical Density at 695 nm		
		Std Ascorbic Acid	*R. apiculata* Extract	AgNPs
1	100	0.27 ± 0.004	0.52 ± 0.002	0.71 ± 0.039
2	200	0.50 ± 0.004	0.79 ± 0.013	0.95 ± 0.008
3	300	0.68 ± 0.004	0.92 ± 0.011	1.12 ± 0.044
4	400	0.90 ± 0.003	1.07 ± 0.001	1.37 ± 0.042
5	500	1.13 ± 0.007	1.27 ± 0.004	1.44 ± 0.004 *

Results were performed in triplicates and expressed as mean± standard error. * One-way ANOVA followed by post Tukey’s test revealed statistically significant differences (*p* ≤ 0.001) between the groups.

**Table 6:** Anti-inflammatory activity of aqueous leaf extract of *R. apiculata* and its synthesized AgNPs.

Sl. No.	Concentration (µg/mL)	Treatment	% Inhibition
1	100	Standard	94.24 ± 1.90 *
2	500	AgNPs	71.65 ± 0.88 *
3	500	*R. apiculata* extract	54.34 ± 3.26 *

Results were performed in triplicates and expressed as mean± standard error. * One-way ANOVA followed by post Tukey’s test revealed statistically significant differences (*p* ≤ 0.001) between the groups.

**Table 7:** Cytotoxicity of aqueous leaf extract of *R. apiculata* and its synthesized AgNPs against L929 cell line.

Samples	Concentration in µg/mL	Percentage of Cell Viability	IC_50_ in µg/mL
*R. apiculata* extract	10	97.44 ± 0.001	47.47
	20	84.03 ± 0.005	
	30	73.55 ± 0.001	
	40	58.65 ± 0.001	
	50	46.81 ± 0.002	
AgNPs	10	98.57 ± 0.005	105.50
	20	92.47 ± 0.001	
	30	90.22 ± 0.001	
	40	83.69 ± 0.005	
	50	77.50 ± 0.005	

Results were performed in triplicates and represented as mean ± standard error.

**Table 8:** Anticancer activity of *R. apiculata* and its synthesized AgNPs against different cancer cell lines.

Samples	Concentration in µg/mL	Percentage of Cell Viability for A375	Percentage of Cell Viability for A549	Percentage of Cell Viability for KB-3-1
*R. apiculata* extract	10	99.72 ± 0.001	99.07 ± 0.003	92.20 ± 0.005
	20	97.35 ± 0.001	92.72 ± 0.001	88.59 ± 0.015
	30	91.05 ± 0.001	87.36 ± 0.002	81.15 ± 0.002
	40	87.75 ± 0.001	83.22 ± 0.003	75.51 ± 0.005
	50	82.69 ± 0.002	73.73 ± 0.002	67.17 ± 0.001
AgNPs	10	86.01 ± 0.005	95.56 ± 0.009	70.95 ± 0.005
	20	65.60 ± 0.002	88.58 ± 0.002	58.12 ± 0.005
	30	54.49 ± 0.002	74.76 ± 0.001	43.16 ± 0.010
	40	44.46 ± 0.005	67.95± 0.004	33.69 ± 0.002
	50	31.84 ± 0.004	56.09 ± 0.010	22.59 ± 0.022

Results were performed in triplicates and represented as mean ± SEM.

**Table 9:** Cell migration of different test samples at different duration.

Sl.No	Test Sample	Duration	Cell Migration in µm
1	Untreated	6 h	2.96
		12 h	2.50
		24 h	2.07
2	Ascorbic acid	6 h	25.47
		12 h	28.03
		24 h	21.74
3	*R. apiculata* extract	6 h	11.63
		12 h	14.58
		24 h	18.74
4	AgNPs	6 h	14.43
		12 h	20.56
		24 h	18.23

**Table 10:** Percentage of wound closure of different test samples.

Sl.No	Test Sample	Percentage of Wound Closure at 24 h
1	Untreated	9.13
2	Standard drug Ascorbic acid	96.26
3	*R. apiculata* extract	75.23
4	AgNPs	82.79

## Data Availability

The data related to this research is included in the results section.

## Sample Availability

Samples of the compounds *R. apiculata* extract and *R. apiculata* AgNPs are available from the authors.
